# Effect of Intergenerational Trends on Parental Child-Rearing Gender Role Attitudes (PCGAs) in Single-Parent Families: A Relative Mediation

**DOI:** 10.3390/bs14070551

**Published:** 2024-06-28

**Authors:** Yunping Song, Mengping Yang, I-Jun Chen, Saba Ponam, Ying Shi

**Affiliations:** 1 School of Education, Soochow University, Wenjing Road, Suzhou Industrial Park (SIP), Suzhou 215123, China; ypsong@stu.suda.edu.cn (Y.S.); 20177218001@stu.suda.edu.cn (S.P.);; 2 Office of Academic Research, Suzhou Early Childhood Education College, Huayuan Road, Xiangcheng District, Suzhou 215123, China

**Keywords:** single-parent children, parental child-rearing gender role attitudes, intergenerational trends, social adaptation, gender role, relative mediation model

## Abstract

Family structures are diverse, with single-parent families being special. Single-parent families have garnered interest regarding their impact on their children’s development in relation to gender roles and social adaptation. This study investigated 532 children from single-parent families (mean age = 14.81, SD = 1.62) and their parents. We collected data on the parental child-rearing gender role attitudes (PCGAs) of grandparents and parents, as well as the gender role and social adaptation of the children. The results revealed four intergenerational trends in PCGAs: progression between generations, undesirability in both generations, desirability in both generations, and retrogression between generations. An ANOVA showed that families with intergenerational desirability tended to have children with the highest gender role and social adaptation scores among the four intergenerational trends, while families with intergenerational undesirability had the lowest. A relative mediation analysis showed that compared to intergenerational undesirable PCGAs, intergenerational progress and intergenerational desirable PCGAs are beneficial for children’s gender traits, and their social adaptation development is also better. The results confirm the positive effect of children’s gender roles on their social adaptation, which suggests that parents should pay attention to children’s gender role education, transform their PCGAs, and create a nurturing environment for children’s gender role development.

## 1. Introduction

In recent years, there has been a noticeable shift in the perception of marriage and childbearing, leading to an increase in the prevalence of single-parent families. According to a survey conducted by the Pew Research Center, single-parent families make up approximately 7% of all families worldwide [1]. In China, single-parent families surpassed 8 million in 2010 and rose to 9 million in 2020. As a result, a significant number of children are being raised in households headed by single parents. The structural deficiencies in such families, stemming from the absence of one parent during a child’s upbringing, may amplify social adaptation issues [2,3,4]. Social adaptation refers to the dynamic process through which individuals achieve harmony with the social environment by conforming to it, self-regulation, or effecting changes within the environment [5]. The social adaptation of an individual is typically manifested in four aspects: interpersonal skills, academic (working) achievement, life skills, and psychological resources [6]. The significant change in family structure experienced by children from single-parent families may lead to increased emotional fluctuations and cognitive shocks, which can impact social adaptation [7]. Well-adapted children can cope with environmental changes and develop strong academic performance as well as interpersonal relationships. Conversely, poorly adapted children may struggle to navigate complex social environments, leading to various psychological and health issues such as anxiety and depression.

Gender roles have been found to influence individuals’ social adaptation significantly. Gender roles refer to a set of gender traits or gender behaviors that individuals acquire through imitation and learning in the process of socialization [8]. According to gender role scale [9], each respondent receives scores for male and female traits. Individuals who score above the median on a subscale that corresponds to their gender and below the median on a subscale that does not correspond to their gender are defined as sex-typed. Those who score below the median on a subscale consistent with their gender and above the median on a subscale inconsistent with their gender are defined as cross-sex-typed. Individuals whose scores on both the male and female trait scales are higher than the median are called androgynous, and those whose scores on both the male and female trait scales are lower than the median are called undifferentiated. Research shows that individuals with androgynous gender roles who possess both masculine and feminine traits tend to exhibit the highest level of social adaptation. Conversely, undifferentiated gender role individuals perform poorly in both masculine and feminine characteristics, such as a lack of strong decision-making ability and patience, showing low levels of social adaptation [10]. Children’s gender development is influenced by the gendered environment created by their parents; from birth, parents often have specific wishes regarding how boys and girls should behave [11], which is reflected in their PCGAs [6]. Traditional PCGAs adherence to socially prescribed gender norms encourages children to develop gender roles that correspond to their biological sex. Conversely, enlightened PCGAs provide children with activities and gender information without gender restrictions, and they are more likely to adopt androgynous gender roles.

According to family system theory [12], parents are influenced by their parents (i.e., grandparents), receiving direct gendered expectations such as “girls should act like girls”. Furthermore, grandparents’ daily behavior, such as career choices and the division of household labor, can subconsciously influence parents’ gender perspectives [13]. When raising their own children, parents’ gender role beliefs inherited from their parents, combined with new life experiences and education, collectively form their PCGAs. Consequently, this may lead to similar PCGAs, more progressive PCGAs, or more conservative PCGAs than the grandparents, establishing a specific trend across generations. Prior research [14] has demonstrated that parenting consistency between grandparents and parents can impact the psychological development of children; however, the patterns of intergenerational trends have yet to be extensively explored. This study will focus on examining the patterns of intergenerational trends of PCGAs between grandparents and parents. It will explore the connection between these intergenerational trends of PCGAs and the gender development as well as social adaptation of children.

### 1.1. PCGAs in Single-Parent Families

Researchers have conducted PCGAs research based on parents’ expectations for their children’s gender roles. There are three main types of classification for PCGAs.

The first is the “stereotype-anti-stereotype” classification; for example, Burge [15] developed a scale for PCGAs, measuring the degree to which parents expect their children to develop gender traits or behaviors consistent with their biological gender (i.e., the degree of sex-typed). Results show the degree of stereotyping in PCGAs. Katz and Walsh focus on how parents react to their children exhibiting cross-sexed gender traits or behaviors, such as boys liking dolls and girls liking cars [16]. This shows the extent to which parents can accept non-stereotype gender traits or behaviors in their children or the degree of anti-stereotyping in PCGAs.

The second classification is “schema-non-schematic” [17,18], where gender schematic parents emphasize that children’s behaviors should conform to their biological gender, similar to sex-typed parenting. In contrast, gender non-schematic parents believe that children should develop both male and female traits, similar to androgynous parenting. This classification is similar to the “traditional PCGAs and non-traditional (egalitarian) PCGAs” [19], where traditional PCGAs require children to adhere to societal gender standards (e.g., boys should be brave, girls should be gentle). On the other hand, non-traditional (egalitarian) PCGAs expect children’s gender traits to include both masculine and feminine characteristics.

The third classification is the four types of PCGAs, divided into masculinization, feminization, androgyny, and undifferentiated [20]. Masculinization refers to expecting children, regardless of their gender, to exhibit more masculine traits and behaviors, and fewer feminine traits and behaviors. Feminization is the opposite, expecting children to exhibit more feminine traits and behaviors and fewer masculine traits and behaviors. Androgynous parenting encourages the development of both masculine and feminine characteristics, and undifferentiated parenting reflects a lack of expectation and neglect of children’s gender development.

**Figure 1:**
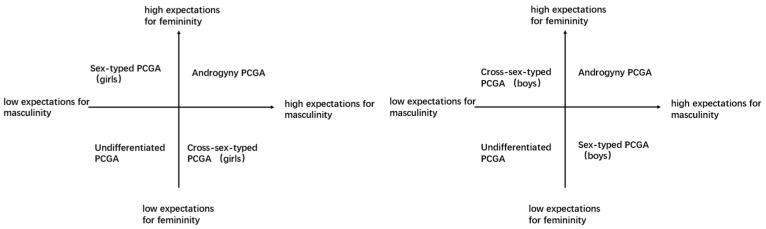
The new four types of PCGAs (the (**left**) for girls and the (**right**) for boys).

From the above classifications, we can observe that parents’ gender expectations based on their children’s biological sex serve as the primary basis for distinguishing PCGAs. The “stereotypical-anti-stereotypical” classification measures PCGAs from the perspective of whether parents’ expected gender roles align with their children’s biological sex (sex-typed PCGAs) or are inconsistent with it (cross-sex-typed PCGAs). The “schematic-non-schematic” classification introduces androgynous PCGAs based on the “stereotypical-anti-stereotypical” classification. Building upon these first two classifications, the third of the “four types of PCGAs” incorporates undifferentiated PCGAs. So far, we have identified four new types of PCGAs, but studies have yet to integrate these. Meanwhile, by combining parents’ expectations of their children’s gender traits with their children’s biological sex, we can indeed derive the same results as the new four types of PCGAs (Figure 1): androgynous, sex-typed, cross-sex-typed, and undifferentiated. These four types comprehensively cover the various possibilities in gender role development, representing a more holistic classification approach.

### 1.2. Intergenerational Trends in PCGAs within Single-Parent Families

According to family system theory [12], grandparents and parents, integral parts of the family system, significantly influence children’s development. The gender roles, expectations, and requirements imposed by grandparents during the upbringing of parents explicitly or implicitly shape parents’ perspectives on gender. As a result, parental PCGAs are inevitably influenced by grandparents. Previous research has indicated that parenting styles within the original familial context can predict individual parenting behavior [21]. There may be both desirable and undesirable intergenerational transmission in cases of intergenerational consistency. Furthermore, parents are influenced by macro systems such as subcultures and societal practices, potentially causing their PCGAs to differ from those of their grandparents. In cases of inconsistency, some parents may adopt more progressive attitudes, signaling intergenerational progress, while others may retain more traditional beliefs, resulting in intergenerational regression. Although there are disparities in intergenerational transmission in cases of intergenerational inconsistency, these distinctions often evade attention in research.

Regarding PCGAs, grandparents and parents can exhibit either consistent or inconsistent PCGAs. Previous research has predominantly focused on consistency or inconsistency between generations [22], lacking in-depth discussions on the scenarios of consistency or inconsistency. Based on the literature review above, we identified sex-typed parenting, cross-sex-typed parenting, androgynous parenting, and undifferentiated parenting as the four new types of PCGAs [9,17]. Sex-typed parenting adheres to traditional gender norms, while androgynous parenting aligns with gender-neutral parenting norms. These two types are considered desirable parenting. Conversely, cross-sex-typed parenting conflicts with traditional gender norms and undifferentiated parenting shows indifference to children’s gender development, thus being regarded as undesirable parenting. Considering this framework, four intergenerational trends may manifest in single-parent families: progression between generations (grandparent undesirable-parent desirable), retrogression between generations (grandparent desirable-parent undesirable), desirability in both generations (grandparent desirable-parent desirable), and undesirability in both generations (grandparent undesirable-parent undesirable).

### 1.3. Intergenerational Trends in PCGAs, Gender Traits, and Social Adaptation

Early studies indicated that children from single-parent families exhibited poorer emotional, cognitive, and social development compared to those from intact families [3]; however, the impact of a single-parent family environment on children’s growth is not uniformly adverse. Several factors, such as parent–child attachment, parenting behaviors, and family functions, can offer protective effects [5,23] and facilitate the social adaptation of children from single-parent households. For instance, parents with traditional PCGAs may believe that girls should be submissive, warm, and sensitive to the needs of others, and they exhibit more visual and physical contact, social initiative, and sensitivity towards girls [24]. These behaviors may help girls become more socially affectionate and promote the development of interpersonal relationships among them. Therefore, PCGAs can function as both protective or risk factors for the social adaptation of children from single-parent families.

According to the specific sensitivity theory [25], interindividual susceptibility variability to protective and risk factors exists. Some individuals display heightened receptivity towards these factors, while others remain unaffected by such influences. When confronted with their parents’ PCGAs, children’s male and female traits may lead individuals to have different reactions, consequently influencing the parenting effect of PCGAs. For example, females’ sensitivity to others’ needs can contribute to a deeper understanding of parental PCGAs [26], thus amplifying their impact. Conversely, the dominant inclinations and tendencies to challenge authority associated with masculine traits may impede a comprehensive understanding of PCGAs, potentially diminishing the influence of such attitudes. Therefore, masculinity and femininity could serve as mediators between PCGAs and children’s social adaptation. Intergenerational trends in PCGAs encapsulate the PCGAs of both grandparents and parents, representing a complex phenomenon. It is plausible that masculine and feminine traits mediate the influence of PCGAs on children’s social adaptation.

### 1.4. Current Study

How single parents parenting their children significantly impacts children’s development. A specific attitude towards parenting, known as PCGAs, is controlled by parents. Establishing the influence of PCGAs on children’s gender roles and social adaptation would offer valuable insights for single parents to provide optimal child-rearing practices. Moreover, within the Chinese context, single-parent families often rely on grandparental resources and the active participation of grandparents in family upbringing [27,28], highlighting the essential role of grandparents in child-rearing. Our data involved three generations (grandparents, parents, and children) to investigate the intergenerational trends of PCGAs and their impact on children’s development. Our hypotheses are as follows:

**Hypothesis** **1.** *The grandparents’ and parents’ PCGAs are expected to be combined in four possible trends: progressing between generations (grandparent undesirable-parent desirable), retrogression between generations (grandparent desirable-parent undesirable), desirability in both generations (grandparent desirable-parent desirable), and undesirability in both generations (grandparent undesirable-parent undesirable).*

**Hypothesis** **2.** *The intergenerational trends on PCGAs would be related to children’s gender traits development. Children in PCGAs families with desirability in both generations score the highest among the four intergenerational trends in gender traits (Hypothesis 2a). Children in PCGAs families with undesirability in both generations score the lowest among the four intergenerational trends in gender traits (Hypothesis 2b).*

**Hypothesis** **3.** *The intergenerational trends on PCGAs would be related to children’s social adaptation. Children in PCGAs families with desirability in both generations score the highest among the four intergenerational trends in social adaptation (Hypothesis 3a). Children in PCGAs families with undesirability in both generations score the lowest among the four intergenerational trends in social adaptation (Hypothesis 3b).*

**Hypothesis** **4.** *Children’s gender traits are mediating intergenerational PCGAs and children’s social adaptation.*

## 2. Materials and Methods

### 2.1. Participants

With the assistance of the local education authorities, we distributed recruitment notices in 15 schools, inviting students and parents to participate in this study. We collected a total of 11,630 sets of student data and 16,589 sets of parent data. By pairing parent–child data based on a student’s ID, 8939 families were ultimately obtained, including 565 from single-parent families; however, 33 questionnaires were excluded due to insufficient response times and excessive consecutive answers [29]. As a result, we were left with 532 valid questionnaires.

The parental sample comprised 532 parents, of which 328 were mothers and 204 were fathers. Among them, 9.77% of the parents originated from single-parent families. When examining household monthly income, 41.54% of parents reported an income of less than RMB 4800, 40.04% reported incomes between RMB 4800 and RMB 9600, 11.84% reported incomes between RMB 9600 and RMB 14,400, and 6.58% reported incomes exceeding RMB 14,400.

The child sample comprised 532 students, including 301 girls and 231 boys, aged 12 to 18 (M = 14.81, SD = 1.62). All of them were from single-parent families. Most students reported that their parents’ parenting styles were democratic (80.08%), while 10.34% reported authoritarian parenting, 6.95% reported neglectful parenting, and 2.63% reported protective parenting. Among the children, 239 were middle school students, with 25.94% in grade 7, 18.23% in grade 8, and 19.55% in grade 9. Additionally, 193 were high school students, with 15.98% in grade 10, 9.96% in grade 11, and 10.34% in grade 12 (Table 1).

### 2.2. Procedure

This study was approved by the Ethics Committee of Soochow University (protocol code: KY20220564B). The data were collected in the larger project “Gender Role Development among Children in Single-Parent Families”. We invited students and their parents from 15 schools to participate in the survey. In collaboration with headteachers, we disseminated the research project brief, informed consent forms, and recruitment notices to all students and parents in written form. The recruitment notice included our contact information, enabling parents and students who wished to volunteer for the project to contact us. Subsequently, we provided the students and their parents with a link to an electronic questionnaire. As a token of appreciation for their participation, both parents and children were offered a small gift.

We conducted surveys for both children and parents, with the children completing the gender role scale, social adaptation scale, and PCGAs scales. The questionnaire results reflected the children’s gender roles, social adaptation, and the parents’ PCGAs. The parents completed PCGAs scales, reflecting the grandparents’ PCGAs, thus forming a dataset that includes three generations. Additionally, the inclusion criteria for the children were as follow: (1) their parents must be divorced, deceased, or separated; (2) the father or mother must be solely responsible for parenting; and (3) they must be under the age of 18.

### 2.3. Measures

#### Parental Child-Rearing Gender Role Attitudes

Parental child-rearing gender role attitudes were measured using an adapted measure from the child-rearing sex role attitude scale [15,19]. The instrument was completed by parents and children separately, with the parental responses reflecting the grandparents’ PCGAs and the children’s responses reflecting the parents’ PCGAs. The instrument included 39 items in the feminized parenting subscale (19 items, e.g., “My parents think I should be gentle and considerate”) and masculine parenting subscale (20 items, e.g., “My parents think I should be strong and brave”). Participants responded using a five-point Likert scale that ranged from 1 (strongly disagree) to 5 (strongly agree). According to Bem’s median classification, individuals’ PCGAs were categorized into androgynous PCGAs (above the median for both the masculine and feminine subscales), undifferentiated PCGAs (below the median for both the masculine and feminine subscales), sex-typed PCGAs (above the median for dimensions consistent with one’s gender and below the median for dimensions inconsistent with one’s gender), and cross-sex-typed PCGAs (above the median for dimensions inconsistent with one’s gender and below the median for dimensions consistent with one’s gender) [9]. Cronbach’s alpha coefficients for each dimension ranged from 0.59 to 0.8514.

### 2.4. Gender Roles

An adaptation of the gender role scale from Bem was used to assess gender roles [9,30]. The instrument consisted of 50 questions, including the masculinity subscale (16 items, e.g., “I am willing to take risks”), femininity subscale (18 items, e.g., “I am affection-ate”), and neutrality subscale (16 items; it served as interference and was not scored). The masculinity subscales were used to measure masculine traits (leadership, masculinity, rationality, and generosity) and the femininity subscale were used to measure femininity traits (empathy, femininity, and thriftiness). The neutral scale is treated as a distractor, not scoring and not measuring any characteristics of the individual. Children rated each item on a seven-point Likert scale ranging from 1 (not at all) to 7 (completely). Higher scores on the masculinity subscale indicate higher masculine traits, higher scores on the femineity subscale indicate higher femineity traits. Cronbach’s alpha was 0.82 for the masculinity subscale and 0.80 for the femininity subscale.

### 2.5. Social Adaptation

Social adaptation was measured using the social adaptation scale [6]. The instrument included 33 items distributed in four dimensions. The dimensions were interpersonal relationships (e.g., “I am good at cooperating with my classmates”), academic achievement (e.g., “I have my study plan and goals”), life skills (e.g., “I can organize my room by myself”), and psychological resources (e.g., “I can quickly forget what I am unhappy about”). The scale was scored on a five-point scale ranging from 1 (never) to 5 (always). Scores were calculated using the mean, and higher values indicated better social adaptation. Cronbach’s alpha coefficient for each dimension ranged from 0.88 to 0.91 [6].

### 2.6. Demographics

Demographic covariates assessed at the intake included children’s grades, age, and gender. Their caregiver gender, duration of singleness, education level, income, household location, and parenting style were also included as covariates. Since parenting style is considered to be significantly correlated with children’s masculinity and femininity [31] and social adaptation [32], we included parenting style as a control variable. We included the question “How do you perceive the parenting style of your parents?” to measure parenting styles. To ensure participants’ understanding of each parenting style, we provided an operational definition for each parenting style [33]. The types of parenting styles include “Democratic (respecting child’s opinions, listening to child’s feelings), Authoritarian (strict discipline, punitive), Neglectful (indifferent to child’s needs), and Protective (indulging the child, unconditional love)”.

### 2.7. Analysis Plan

In this research, missing data were not a concern, as electronic questionnaires were provided, and all questions were considered compulsory. The analysis included three generations, encompassing grandparents’ PCGAs, parents’ PCGAs, and children’s traits and social adaptation.

We used SPSS 24.0 and the SPSS macro (version 3.3) developed by Hayes and Preacher [34]. First, we categorized intergenerational trends in PCGAs by pairing grandparents’ PCGAs with parents’ PCGAs. Second, we used an ANOVA to calculate the children’s gender traits and social adaptation across families with different intergenerational trends in PCGAs. After this, we used a correlation analysis to calculate the correlation between independent, dependent, and covariate variables to identify control variables for subsequent modeling. Finally, we set a relative mediation model to test the mediating role of children’s gender traits in the intergenerational trends in PCGAs and children’s social adaptation.

We chose relative mediation model [34,35] because the independent variable “PCGAs” was categorical.

According to the process of multi-categorical mediation analysis [35]:

(1) k levels of the independent variables were coded (k-1) by selecting appropriate reference levels. In this study, we coded the undesirable PCGAs in both generations as the reference group, D1 referring to intergenerational progress PCGAs, D2 referring to intergenerational retrogressive PCGAs, and D3 referring to desirability PCGAs in both generations.
(2) Conduct a total mediation analysis to obtain the overall mediation effect, which represents the impact of the independent variable, X, on the dependent variable, Y. If the overall mediation effect is significant, it indicates that at least one of the k-1 relative mediation effects is significant. If the overall mediation effect is not significant, it means that there is no relative mediation effect.
(3) On the basis of step two, conduct a relative mediation analysis to obtain the relative direct effect and the relative indirect effect. The relative direct effect quantifies the difference on the dependent variable, Y, caused by the difference in the independent variable, X between different groups. The relative indirect effect quantifies the difference in the dependent variable, Y, caused by independent variable, X, in differences between groups and then through the mediator, M. The sum of the relative direct effect and the relative indirect effect is the relative total effect. This step can clarify which the relative mediation effects are significant.
(4) Report the significance of the relative mediation effect. Hayes and Preacher recommend using the bootstrap method to judge the significance of the relative mediation effect, by conducting 5000 repeated samples and estimating the 95% confident interval. To test whether the 95% confidence interval of a*b contains 0. If it does not contain 0, indicating the significant of mediation [34].

## 3. Results

### 3.1. Intergenerational Trends in PCGAs

To explore the intergenerational trends in PCGAs, the study categorized the four types of PCGAs into two groups: desirable PCGAs (including androgynous PCGAs and sex-typed PCGAs) and undesirable PCGAs (undifferentiated PCGAs and cross-sex-typed PCGAs). We combined grandparents’ and parents’ PCGAs into four intergenerational trends (Table 2): progressing between generations, undesirability in both generations, desirability in both generations, and retrogression between generations. As shown in Table 2, 27.63% of families were intergenerationally desirable PCGAs, 21.05% were intergenera tionally undesirable PCGAs, 33.64% were intergenerationally retrogressive PCGAs, and 17.67% were intergenerationally progressive PCGAs.

### 3.2. Children’s Gender Traits and Social Adaptation in Intergenerational Trends in PCGAs

Table 3 shows the chi-square analysis results testing children’s social adaptation and gender traits across the four trends of intergenerational PCGAs. The findings indicated significant differences (*p* < 0.05) in social adaptation and gender traits among the four trends. According to a post-hoc analysis, children from intergenerational desirable families had the highest masculine and feminine trait scores, significantly higher than those in other intergenerational PCGAs families. Children in intergenerational progressive families had substantially higher masculine and feminine traits than those in intergenerational undesirable families. The masculinity of children in intergenerational progressive families was significantly higher than that of children in intergenerational retrogressive families, whereas there was no significant difference in femininity. And no significant difference was observed in femininity between intergenerational undesirable and intergenerational retrogressive families.

Children from intergenerational desirable families had the highest social adaptation scores, significantly higher than other intergenerational PCGAs trend families. Conversely, children from intergenerational undesirable families had the lowest social adaptation scores, substantially lower than other intergenerational PCGAs trend families. Children’s social adaptation in intergenerational progressive families was significantly higher than in intergenerational undesirable and retrogression families. According to the research findings, no significant difference was observed in children’s social adaptation between intergenerational undesirable and retrogressive families.

### 3.3. Mediation Analyses

Table 4 displays all variables’ means, standard deviations, and intercorrelations. The bivariate correlations revealed that intergenerational trends in PCGAs were significantly) correlated with children’s masculine traits, feminine traits, and social adaptation. Additionally, the correlations between children’s social adaptation and feminine as well as masculine traits were significant.

Moreover, children’s masculine traits were significantly correlated with family income (*p* < 0.05), while children’s feminine traits were significantly correlated with parenting style (*p* < 0.01) and children’s gender (*p* < 0.05). Children’s social adaptation was significantly correlated with parenting style (*p* < 0.01). Consequently, family income, parenting style, children’s gender, and children’s grades were incorporated into the mediation analysis as control variables.

We conducted an overall mediation analysis test to analyze the increase in *ΔR*^2^ after adding k-1 Di variables to the model. The results demonstrated that the overall mediation analysis result was significant, with Δ*R*^2^ = 0.046, *F* = 13.507 (*p* < 0.001). The result suggested that at least one relative mediating effect was significant, therefore, the mediating effect of gender traits between intergenerational trends in PCGAs and children’s social adaptation was confirmed, enabling a relative mediation analysis.

**Figure 2:**
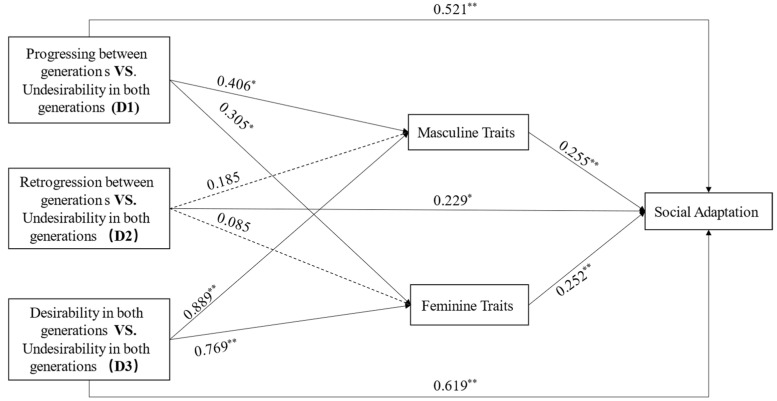
Multiple mediation model. All path coefficients are standardized coefficients, * sig. at *p* < 0.05, ** sig. at *p* < 0.01, based on the bootstrapping of 5000 subsamples.

The results of the relative direct effect analysis (Figure 2) showed that intergenerational trends in PCGAs had significant direct effects on children’s social adaptation. Children from intergenerational progressive families exhibit better social adaptation than those from intergenerational undesirable families (*β* = 0.521, *p* < 0.000). Children’s social adaptation in intergenerational retrogressive families was better than in intergenerational undesirable families (*β* = 0.229, *p* < 0.05). Furthermore, children from desirable intergenerational families’ social adaptation was superior to that of those from undesirable families (*β* = 0.619, *p* < 0.000).

The results of the relative indirect effect indicated that children’s masculinity and femininity be affected by specific intergenerational trends in PCGAs and then influence children’s social adaptation (Table 5, Figure 2). Specifically, in comparison to intergenerational undesirable families, children from intergenerational progressive families had higher scores for masculine traits (*β* = 0.406, *p* < 0.001) and better social adaptation (*β* = 0.103, *p* < 0.000), along with higher feminine traits (*β* = 0.305, *p* < 0.05) and better social adaptation (*β* = 0.077, *p* < 0.000). Similarly, compared to children from intergenerational undesirable families, those from intergenerational desirable families had higher score in masculine traits (β = 0.889, *p* < 0.000) and better social adaptation (*β* = 0.226, *p* < 0.000), as well as higher scores in feminine traits (*β* = 0.769, *p* < 0.000) and better social adaptation (*β* = 0.194, *p* < 0.000). However, there was no significant difference in children’s masculine and feminine traits between intergenerational retrogressive families and intergenerational undesirable families (*β* = 0.185, *p* = 0.126; *β* = 0.085, *p* = 0.484).

## 4. Discussion

It is widely acknowledged that androgynous gender roles are conducive to children’s social adaptation; however, in real life, children are confronted with a more complex gendered environment, and they do not always develop androgynous gender roles. The PCGAs of grandparents and parents constitutes the family environment for the gender development of children. This raises the question whether: what forms do they take, and can they guide single-parent children to develop androgynous gender roles and good social adaptation? This study examined parents’ and grandparents’ intergenerational trends in PCGAs using Bem’s gender role classification [9,17]. Then, this study explored the relationship between these intergenerational trends in PCGAs and children’s gender roles as well as social adaptation.

### 4.1. Intergenerational Trends in PCGAs in Single-Parent Families

As hypothesized, four kinds of intergenerational trends in PCGAs are present in single-parent families: progressing between generations, undesirability in both generations, desirability in both generations, and retrogression between generations. Among them, the proportion of intergenerational desirable families (27.63%) is greater than that of intergenerational undesirable families (21.05%) in single-parent families. This may be due to altruistic tendencies in parenting [36], where parents tend to pass on mainstream and socially accepted values to children to help them develop. Intergenerational desirable PCGAs includes androgynous PCGAs and sex-typed PCGAs. Androgynous PCGAs emphasize the cultivation of androgynous traits, which aligns with modern gender concepts. Sex-typed PCGAs emphasize the alignment of social gender and biological sex, which aligns with traditional cultures, therefore, intergenerational desirable PCGAs are more likely to be passed on because they conform to modern or traditional gender concepts that society respects or affirms. On the other hand, intergenerational undesirable PCGAs includes undifferentiated PCGAs and cross-sex-typed PCGAs. Undifferentiated PCGAs manifests indifference to children’s gender development, contradicting the androgynous gender perspective. Cross-sex-typed PCGAs manifest high expectations for children’s heterosexual traits, contradicting the traditional gender perspective. This is also not conducive to the development of children’s gender roles, just like the negative connotations attached to “effeminate men” and “masculine women” in society. Based on socialization theory, attitudes and values that are socially undesirable (i.e., not accepted by the majority of society) are less likely to be passed on [37], thus, the proportion of intergenerationally undesirable PCGAs is relatively small.

The proportion of intergenerational retrogressive families (33.64%) was higher than that of intergenerational progressive families (17.67%). This may be because all parents in this study were single-parent families; they often have limited time for gender parenting since they have to balance a career and raise a family at the same time [38], resulting in many undifferentiated PCGAs. On the other hand, it has been observed that single parents may develop excessive cross-sex-typed expectations of their children due to the unique demands and challenges they face in their daily lives [39]. For instance, single mothers may find themselves shouldering both male and female responsibilities, leading them to expect their daughters to assume more male roles, resulting in more intergenerational retrogressive PCGAs.

### 4.2. Intergenerational Trends in PCGAs and Children’s Gender Traits in Single-Parent Families

Children from intergenerational undesirable families exhibit underdeveloped masculine and feminine traits (Hypothesis 2b). This may be due to the influence of both grandparents and parents holding undesirable PCGAs. In families where grandparents and parents provide stable and consistent undesirable gender information, children may be guided in an undesirable manner, which can result in undeveloped gender traits. Furthermore, co-parenting is often observed in single-parent families [40]. When both grandparents and parents use undesirable PCGAs, the effects of undesirable PCGAs achieve intergenerational accumulation [41], which makes children’s gender traits develop the lowest scores of the four family trends.

Children from intergenerational retrogressive families exhibit no progress in masculinity and femininity compared to those from intergenerational undesirable families. In intergenerational retrogressive families, children receive desirable PCGAs from their grandparents but are exposed to undesirable PCGAs by their parents. In contemporary Chinese parenting practices, parents assume the principal responsibility for children’s development and have the right to make decisions about children’s development. Thus, their undesirable PCGAs guides the inappropriate development of children’s gender roles. While grandparents enter their families as “helpers” and take on a large amount of the physical upbringing and daily care of children, they are marginalized in the decision making of family affairs [42]. Furthermore, they focus primarily on providing daily care and ensuring growth and safety rather than actively engaging in cognitive, personality, and social development. Consequently, grandparents’ desirable PCGAs does not significantly influence the direction of children’s gender traits. In conclusion, parents play a significant and direct role in their children’s development, even when co-parenting with grandparents.

Masculine traits were higher in intergenerationally progressive families than in intergenerationally retrogressive and undesirable families, but feminine traits were higher in intergenerationally undesirable families. In intergenerational progressive families, along with the desirability of the family’s PCGAs, the development of masculinity is prioritized. This may be related to the culture in China, where male privilege has deep cultural roots. They preferred sons over daughters because they believed that men were responsible for the continuation of the family [43] and that males had “earning power” over females [44]. Such expectations may have reinforced parents’ preferences for sons and placed a higher value on masculine traits, leading to their higher expectations of children’s masculine traits.

Children from desirable intergenerational families exhibit the highest scores for masculine and feminine traits (Hypothesis 2a). In such families, both grandparents and parents have desirable PCGAs, which would supply a supportive family environment for children’s gender development. Moreover, grandparents emphasize repeated desirable gender messages, reinforcing desirable gendered parenting, and its effect achieves intergenerational accumulation [41].

### 4.3. Intergenerational Trends in PCGAs and Children’s Social Adaptation in Single-Parent Families

The social adaptations of children in intergenerational undesirable families are often found to be lowest compared to those in other families (Hypothesis 3b). In such families, grandparents’ and parents’ PCGAs are cross-sex-typed PCGAs or undifferentiated PCGAs. Cross-sex-typed PCGAs encourages children to develop gender traits that are contrary to tradition. However, children who violate traditional gender norms often experience negative evaluation [45], higher victimization and exclusion [46], and less acceptance by their peers during social adaptation [47]. Moreover, undifferentiated grandparents and parents neither encourage nor restrict children’s activities and gender development. Combined with the lack of family resources often experienced in single-parent families [38,48], children in intergenerational undesirable families exhibit lower levels of social adaptation than those from other families. Children from intergenerational progressive families exhibit higher social adaptation levels than those from intergenerationally retrogressive and undesirable families.

Children in intergenerational progressive families receive positive family support and guidance, encouraging the development of traditional androgynous gender traits. Children from the families feel accepted and supported by others, enabling them to build a more supportive social system and improve their interactions with external environments [49]. In addition, children from single-parent families who receive support from social systems can better fulfill multiple social roles and maintain a work-life balance, facilitating their social adaptation even when living in a single-parent household.

Children in intergenerational desirable families have the highest scores of social adaptations (Hypothesis 3a). In such families, both grandparents and parents hold desirable PCGAs. Parents and grandparents provide consistent, positive guidance and support for children’s gender development. Additionally, grandparents and parents complement each other’s parenting, with grandparents providing desirable parenting when parents are busy with work and parents providing correct parenting when grandparents may not have the energy to do so. This comprehensive and complete desirable parenting in intergenerational desirable families leads to intergenerational accumulation of parenting effects, ultimately resulting in children’s better social adaptation.

### 4.4. The Mediating Role of Children’s Gender Traits

Compared with intergenerational undesirable families, intergenerational progressive families, and intergenerational desirable families have relative mediating effects. The results showed that children’s masculinity and femininity traits were better and that social adaptation development was also better in intergenerational progressive and intergenerational desirable families (Hypothesis 4 is partially supported). This may be because, in both intergenerational progressive families and intergenerational desirable families, parents hold desirable PCGAs, and the development of children’s gender traits is supported, which is conducive to children’s societal adaptation; however, in intergenerational undesirable families, both grandparents and parents hold negative PCGAs, in which families’ children’s gender traits are not fully developed. As a consequence, children’s social adaptation is also inadequate.

Compared with intergenerational undesirable families, intergenerational retrogressive families have no relative mediating effect but have a significant direct impact on children’s social adaptation. It may be that because of intergenerational retrogression, the grandparent’s PCGAs in those families are desirable (that is, androgynous or sex-typed), manifested as support and encouragement for children’s development. Grandparents provide children with direct encouragement or indirect resource transfer to achieve education or socioeconomic status [50]. The results are consistent with previous studies, suggesting the auxiliary role of grandparents in co-parenting [51].

## 5. Limitations and Future Research Directions

The current study has some limitations, leaving opportunities for future research. First, the current study categorizes androgynous and sex-typed PCGAs as desirable while considering undifferentiated and cross-sex-typed PCGAs as undesirability. however, androgynous PCGAs are regarded as a more comprehensive and healthier PCGAs than sex-typed PCGAs. Combining them into one category may weaken the effect of androgynous PCGAs or exaggerate the impact of sex-typed PCGAs. Similarly, the differences between cross-sex-typed PCGAs and undifferentiated PCGAs are ignored when they are both labeled undesirable. Thus, the current classification describes the intergenerational trends in PCGAs. Future studies can develop new criteria with which to capture more intergenerational trends in family parenting attitudes, resulting in a more accurate depiction of intergenerational PCGAs.

Additionally, cross-sex-typed PCGAs refer to situations where parents hold higher expectations for gender traits that do not align with their children’s biological sex and lower expectations for those that align with children’s biological sex; however, in practice, cross-sex-typed PCGAs may include more complex situations. For example, androgynous PCGAs may occur when fathers expect their daughters to be strong and brave if they are too soft and dependent. Nevertheless, distinguishing between these situations can be challenging on a large-scale questionnaire. This highlights the need for more detailed research on cross-sex-typed PCGAs, including case studies and qualitative research.

Furthermore, this study employs a mediation analysis using a multi-categorical independent variable approach recommended by Hayes and Preacher [34]. Specifically, the study calculates relative mediating effects, relative direct effects, and total effects for intergenerational trends in PCGAs, with intergenerational undesirable PCGAs serving as the reference variable; however, the choice of reference level can influence the relative mediation results. Future studies may consider treating intergenerational trends in PCGAs as a continuous variable with which to examine the relationship using a traditional mediation analysis.

## 6. Conclusions

The gender roles and social adaptation of single-parent children have seen wide interest, due to the special nature of this family structure. Many factors have been proven to be beneficial for the development of single-parent children, providing protective effects. If it can be proven that intergenerational PCGAs have protective effects, they may have practical implications for parenting. The study observed four intergenerational trends in PCGAs in single-parent families: progressing between generations, undesirability in both generations, desirability in both generations, and retrogression between generations. This step highlights the importance of grandparent co-parenting in single-parent families and expands existing research on this topic. Furthermore, the study suggests that the impact of grandparental coparenting on children’s development was cumulative when grandparents’ and parents’ PCGAs were consistent; however, when PCGAs were inconsistent, parents’ PCGAs took precedence in determining children’s development, while grandparents’ PCGAs played a complementary role. These findings emphasize the importance of parents or grandparents providing normal guidance. In particular, they highlight the importance of grandparents and parents maintaining normal and consistent attitudes towards PCGAs. This consistency not only helps to build a stable and positive environment for children’s growth but is also crucial for single-parent children to achieve gender role development and social adaptation.

## Figures and Tables

**Table 1:** Demographic information of participants.

	Parents		Children	
	N	Percentage	N	Percentage
Gender				
Father	204	38.35%	231	43.42%
Mother	328	61.65%	301	56.58%
From Single-Parent Family				
Yes	52	9.77%	532	100%
No	480	90.235	0	0
Monthly Household Income				
RMB 4800	221	41.54%		
RMB 4800~9600	213	40.04%		
RMB 9600~14,400	63	11.84%		
Above RMB 14,400	35	6.58%		
Parenting style				
Democratic			426	80.08%
Authoritarian			55	10.34%
Neglectful			37	6.95%
Protective			14	2.63%
Grade				
Grade 7			138	25.94%
Grade 8			97	18.23%
Grade 9			104	19.55%
Grade 10			85	15.98%
Grade 11			53	9.96%
Grade 12			55	10.34%

**Table 2:** Classification of the intergenerational trends in PCGAs (N = 532).

Intergenerational Trends of PCGAs	Grandparents to Parents	N (%)
Progressing between generations	Undesirability to Desirability	94 (17.67%)
Undesirability in both generations	Undesirability to Undesirability	112 (21.05%)
Desirability in both generations	Desirability to Desirability	147 (27.63%)
Retrogression between generations	Desirability to Undesirability	179 (33.64%)

**Table 3:** Comparison of gender traits and social adaptation by intergenerational trends in PCGAs.

Intergenerational PCGAs	Children’s Gender Trait				Children’s Social Adaptation	
	Masculine Traits		Feminine Traits			
	M	SD	M	SD	M	SD
Progressing between generations	5.00	0.99	5.08	0.98	3.97	0.61
Undesirability in both generations	4.55	1.00	4.78	1.03	3.38	0.67
Retrogression between generations	4.77	0.88	4.86	0.85	3.65	0.74
Desirability in both generations	5.47	0.95	5.53	0.90	4.20	0.65
Total	4.97	1.00	5.08	0.97	3.82	0.74
F	22.505 ***		18.032 ***		34.517 ***	
η2 partial	0.113		0.093		0.164	

*** Correlation is significant at the 0.001 level (two-tailed).

**Table 4:** Descriptive statistics and bivariate correlations between study variables.

	1	2	3	4	5	6	7	8	9	10	11	12	Means	SD
1 Caregiver gender	1													
2 Family income	−0.05	1												
3 Parenting style	−0.06	−0.05	1											
4 Parental education	0.07	0.41 **	−0.08	1										
5 Durations of singleness	0.02	0.12 **	−0.07	0.11 *	1									
6 Children’s gender	0.00	−0.07	−0.09 *	−0.03	0.03	1								
7 Children’s age	0.03	−0.08	−0.07	−0.10 *	0.06	−0.02	1							
8 Children’s grade	0.15	0.02	−0.05	−0.09	0.11	−0.09	0.60 **	1						
9 Intergenerational PCGAs	0.04	0.01	0.01	0.04	−0.07	0.01	0.01	−0.12	1					
10 Children’s masculine	0.03	0.10 *	−0.08	0.05	−0.01	−0.05	−0.04	0.02	0.19 **	1			4.98	1.00
11 Children’s feminine	−0.02	0.02	−0.12 **	0.03	−0.05	0.10 *	0.01	0.06	0.17 **	0.79 **	1		5.08	0.97
12 Children’s social adaptation	0.01	0.07	−0.23 **	0.04	0.01	−0.03	−0.06	−0.11	0.15 **	0.54 **	0.53 **	1	3.82	0.74

* Correlation is significant at the 0.05 level (two-tailed), ** correlation is significant at the 0.01 level (2-tailed).

**Table 5:** Multiple mediation model path coefficients.

	Path	Indirect Effect	SE	Boot LLCI	Boot ULCI
Progressing between generations VS. Undesirability in both generations (D1)	X→M1→Y	0.103	0.045	0.027	0.200
	X→M2→Y	0.077	0.042	0.015	0.169
	Relative total mediation effect	0.383	0.082	0.222	0.544
	Relative total effect	0.515	0.938	0.331	0.700
Retrogression between generations VS. Undesirability in both generations (D2)	X→M1→Y	0.047	0.035	−0.014	0.124
	X→M2→Y	0.022	0.033	−0.043	0.090
	Relative total mediation effect	0.168	0.073	0.024	0.312
	Relative total effect	0.219	0.085	0.053	0.385
Desirability in both generations VS. Undesirability in both generations (D3)	X→M1→Y	0.226	0.647	0.114	0.366
	X→M2→Y	0.194	0.056	0.092	0.310
	Relative total mediation effect	0.455	0.080	0.298	0.612
	Relative total effect	0.764	0.088	0.591	0.937

Note: LLCI refers to the lower limit of confidence interval, and the ULCI refers to the upper limit of confidence interval. They represent the likelihood that the true value of a parameter falls within this range. By checking whether the value between the LLCI and ULCI includes 0, researchers can determine the significance of a path, where the absence of 0 indicates significance.

## Data Availability

The original data presented in the study are openly available in Harvard Dataverse (https://doi.org/10.7910/DVN/EKGR4N (accessed on 15 September 2023)).
